# Relationship between kisspeptin-10, neurokinin B and dynorphin A in the course of normal and delayed puberty in ewes

**DOI:** 10.2478/jvetres-2026-0010

**Published:** 2026-03-02

**Authors:** Natalia Szysiak, Urszula Kosior-Korzecka, Monika Greguła-Kania, Krzysztof Patkowski, Mateusz Fila, Andrzej Junkuszew

**Affiliations:** Sub-Department of Pathophysiology, Department of Preclinical Veterinary Sciences, Faculty of Veterinary Medicine, University of Life Sciences in Lublin, 20-033 Lublin, Poland; Department of Animal Breeding and Agricultural Consulting, Faculty of Animal Sciences and Bioeconomy, University of Life Sciences in Lublin, 20-950 Lublin, Poland

**Keywords:** dynorphin A, ewe, kisspeptin, longitudinal study, neurokinin B, puberty disorders

## Abstract

**Introduction:**

Kisspeptin (KiSS), neurokinin B (NKB) and dynorphin A (Dyn A) participate in the neuroendocrine regulation of reproductive system development and functioning; however, their roles in the onset of sexual maturity and pathomechanism of delayed puberty have not yet been fully elucidated. The aim of the study was to determine changes in the kisspeptin-10 (KiSS-10), NKB and Dyn A concentration in blood plasma collected from ewe lambs during puberty in relation to the first ovulation time, and to examine the relationship between these neuropeptides.

**Material and Methods:**

Twenty-four ewe lambs were divided into two groups: from single (S) pregnancies and from twin (T) pregnancies. To determine the KiSS-10, NKB and Dyn A concentration with ELISA, blood was collected from all sheep at 4, 5, 6, 7, 8, 9 and 10 months of age. Singletons and twins had their first ovulations at 8 and 10 months of age, respectively.

**Results:**

Changes in the KiSS-10 and NKB concentrations were positively correlated with age in both S (r = 0.86) and T ewes (r = 0.89). A negative correlation was found between KiSS-10 and Dyn A (r = –0.55 and –0.98, for S and T, respectively) and between NKB and Dyn A (r = –0.89 and –0.94, for S and T, respectively).

**Conclusion:**

The initiation of ovarian activity is associated with concurrent increases in plasma KiSS-10 and NKB concentrations with age and reduced Dyn A concentrations in both groups. Our results show that these neuropeptides may regulate the timing of reproductive activity onset in sheep.

## Introduction

The pathomechanism of delayed puberty due to various aetiological factors has not been definitively elucidated. A delay in ewe puberty, which normally occurs in the first year of life, prevents reproductive activity at the right time and thus reduces reproductive performance. Evidence from other mammalian species point to associations with hormonal and metabolic disorders (1, 5, 24, 28). One of the causes of delayed puberty in lambs is low birth weight. It is observed in ewes from twin pregnancies and the offspring of mothers with a high body weight (9, 22, 23). Furthermore, achieving sexual maturity *via* activation of the hypothalamic–pituitary–ovarian (HPO) axis depends on the adequacy of fat reserves contained in subcutaneous and visceral adipose tissue as well as on key metabolic signals, *e.g*. an adequate plasma leptin concentration (34). Leptin, a peptide released from white adipose tissue and binding to the long-form (OB-Rb) and short-form (OB-Ra) obesity receptors in the hypothalamus, mediates the activation of GnRH neurons. The activation of these neurons induces gonadoliberin release, leading to increased LH and FSH secretion by the anterior pituitary gland (25, 34). In the hypothalamus, this effect of leptin is mediated by neuronal agents, such as kisspeptins (KiSSs) (2, 8, 17).

KiSSs, belonging to the arginine-phenylalanine amide (RF-amide) peptide family, along with other neuropeptides such as neurokinin B (NKB) from the tachykinin family, and dynorphin (Dyn), representing the family of endogenous opioid peptides, are produced by KNDy (kisspeptin, neurokinin B, and dynorphin) neurons in the arcuate nucleus (ARC) of the hypothalamus (15). A key role is played by KiSSs, NKB and Dyns in the endocrine regulation of the reproductive system during puberty. The function of NKB in puberty in sheep is not direct stimulation of gonadotropin-releasing hormone (GnRH) neurons, because these neurons lack the neurokinin 3 receptor for NKB (NK3R) which ARC KNDy neurons have. It is believed that stimulation of GnRH/LH secretion by NKB occurs indirectly *via* activation of the ARC KNDy neurons, which leads to increased KiSS production (3, 14, 20). A kisspeptin interacts with its receptor on GnRH neurons located in the preoptic area (POA) and the ARC of the hypothalamus. This interaction enables the pulsatile release of GnRH, which subsequently induces the anterior pituitary to secrete gonadotropins (15). It is known that the lack of NKB and NK3R causes hypogonadotropic hypogonadism and failure to mature sexually in humans (29). As reported by Billings *et al*. (4), intraventricular application of an NKB analogue (senktide) in mature ewes caused surge-like secretion of LH. Additionally, intravenous application of KiSS-10 in concentrations of 0.5, 1 and 2 mg increased LH secretion in prepubertal female Small-tail Han Sheep (31). The role of Dyns, in turn, in puberty is much less well explained than the role of KiSS or NKB. It is known that the receptor for Dyns, *i.e*. the κ opioid receptor (KOR), is located in KNDy and GnRH neurons in many animal species, including sheep (33). In studies conducted by Lopez *et al*. (12), it was shown that a Dyn plays an important role in inhibiting the release of GnRH, which results in the inhibition of pulsatile LH secretion in prepubertal ewe lambs. However, previous research demonstrated that Dyn A as well as KiSS-10 and NKB can directly stimulate gonadotropin secretion from ovine anterior pituitary cells *in vitro* (26). The aim of the present study was to determine the changes in the concentration of KiSS-10, NKB and Dyn A in blood plasma collected from ewe lambs during puberty in relation to the time of the first ovulation. The experiment included sheep coming from single and twin pregnancies, the latter being predisposed to delayed puberty.

## Material and Methods

### Experimental design

The protocol of the experimental design and all procedures were approved by the Local Ethics Committee for Animal Experimentation in Lublin (Licence No. 65/2023). Twenty-four female ewe lambs of the Polish Uhruska breed housed at the Didactic-Experimental Station of Sheep and Goat Breeding in Bezek were used in the study. The first group consisted of lambs from single pregnancies (n = 13), and the second group consisted of lambs from twin pregnancies and thus predisposed to delayed puberty (n = 11). Weight gain was monitored in all sheep from birth to the age of 10 months. The study was carried out from May to November under natural light and in normal temperature. The animals were kept in the indoor system from January to mid-May and from September to December, and in the indoor-outdoor system from mid-May to early September in conditions that ensured freedom of movement. After weaning, the sheep were fed once a day with complete age-appropriate farm feed for sheep comprised of hay, haylage and concentrate and had unlimited access to water. The animals were kept in identical nutritional and environmental conditions to those in a commercial herd and their nutritional needs were met according to the French Institut National de la Recherche Agronomique feeding system (according to their physiological status). The lambs lived in a common sheepfold with their mothers from birth to weaning and were only selected for the experiment when they reached the age of 4 months. After being divided into the two experimental groups, they were transferred to another flock 10 days before blood collection began in order to adapt to the new environment. The sheep were weighed using an electronic scale every two weeks from birth to 10 months of age, and weight gain was recorded. From 6 to 10 months, the activity of the ewes’ ovaries was monitored laparoscopically for the presence, number and diameter of follicles or the presence of corpora lutea. To determine the concentration of NKB, Dyn A and KiSS-10 after the ewes had reached 4, 5, 6, 7, 8, 9 and 10 months of age, 8 mL of blood was collected from the external jugular vein with the use of intravenous cannulas every 15 min for 2 h. After the procedure, the samples were centrifuged for 20 min at 1,000 rpm and stored at -20°C until testing. Plasma NKB, Dyn A and KiSS-10 concentrations were analysed by ELISA using species-specific antibodies in a Sheep NKB ELISA Kit and Sheep Dyn ELISA Kit (both from Sunred Biological Technology, Shanghai, China) and a KiSS-1 (112–121) Amide/Kisspeptin10/Metastin (45–54) Amide EIA (enzyme immunoassay) Kit (Phoenix Pharmaceuticals, Burlingame, CA, USA) (18).

### Statistical analysis

The results were calculated using Statistica 13.3 PL (TIBCO, Palo Alto, CA, USA) and expressed as a mean and standard deviation (x ± S.D.). Comparisons between the parameters of ewe lambs coming from single pregnancies and those from twin pregnancies were performed using the analysis of variance and paired *t*-tests. Differences with a probability of P ≤ 0.05 were considered significant. Pearson linear correlation coefficients were calculated to assess the relationships between the analysed variables, namely NKB, KiSS-10 and Dyn A secretion.

## Results

### Activity of ovaries

The singleton ewes ovulated for the first time at 8 months of age. This was about 7–8 weeks earlier than the twin ewes, in which the first ovulations were observed at 10 months of age. The first ovulation was ascertained from the formation of the first corpora lutea.

### Changes in body weight of singleton and twin ewes

The mean body weight of ewes coming from single pregnancies was higher than the weight of ewes from twin pregnancies during the whole experimental period (Table 1). In both singletons and twins, it reached the highest value in the 10^th^ month. The highest percentage difference in the mean body weight between the groups was observed at birth, and this difference decreased from birth to the ninth month, when it was the lowest.

**Table 1.** Mean body weight of singleton and twin Polish Uhruska ewes and differences in that weight at age points up to the 10^th^ month

		Age (months)							
		0	4	5	6	7	8	9	10
Mean body weight ± SD (kg)	Singletons	5.60 ± 0.6	32.16 ± 4.6	34.69 ± 4.1	35.26 ± 4.2	37.57 ± 4.2	37.96 ± 3.9	39.27 ± 3.8	40.96 ± 3.9
	Twins	4.70 ± 0.5	29.51 ± 5.1	31.86 ± 3.2	33.11 ± 2.9	35.97 ± 2.9	36.68 ± 3.1	38.23 ± 2.7	39.15 ± 2.6
Difference in mean body weight (%)		16.07	8.24	8.16	6.10	4.26	3.37	2.65	4.42

### Concentration of kisspeptin-10 in blood plasma

**Fig. 1.**
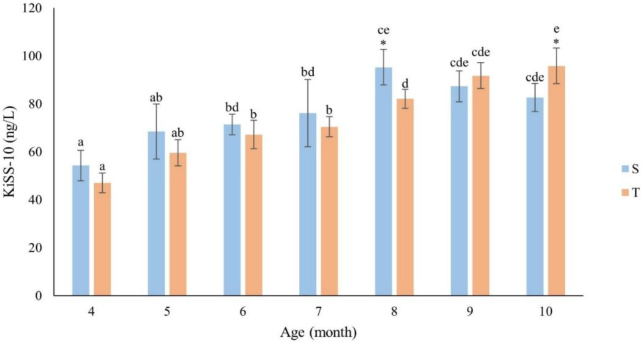
Concentration of kisspeptin-10 (KiSS-10) (ng/L) by month of life of singleton (S – n = 13) and twin (T – n = 11) ewes. The values presented in the figure are mean (±SD) concentrations in the plasma collected monthly every 15 min for 2 h. * – statistically significant difference between S and T (P-value ≤ 0.05); a, b, c, d and e – mean values in a particular month marked with different letters differ significantly (P-value ≤ 0.05)

The KiSS-10 concentration in blood plasma was dependent on the month of life of the ewes (Fig. 1). In S ewes, the KiSS-10 levels increased until the eighth month, with a significant (P-value ≤ 0.05) rise from the seventh month. This maximal value was significantly higher (P-value ≤ 0.05) than those in the period from the fourth to seventh month. This was related to the onset of ovarian activity in this group. In T ewes, a continuous increase in the KiSS-10 levels was observed throughout the experimental period. The highest mean plasma concentration of KiSS-10 in multiple-birth ewes was confirmed after the end of the 10^th^ month, but this concentration (95.8 ng/L) barely exceeded the concentration in the singletons at the end of the 8^th^ month (95.28 ng/L). This correlated with the initiation of ovarian activity, as it did for the singletons. A positive relationship was shown between the KiSS-10 concentration and the age (r = 0.84 and r = 0.99 for S and T, respectively) of the ewes. There was a significant (P-value ≤ 0.05) difference in plasma KiSS-10 concentrations between the groups after the 8^th^ and 10^th^ months of life, associated with the negative correlation noted between concentration and age for S animals in this period (Fig. 1).

### Concentration of neurokinin B in blood plasma

**Fig. 2.**
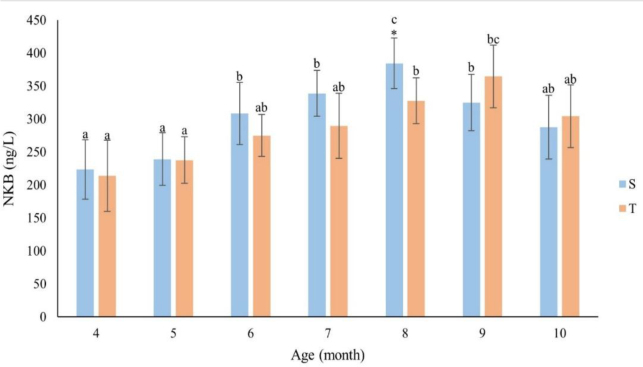
Concentration of neurokinin B (NKB) (ng/L) by month of life of singleton (S – n = 13) and twin (T – n = 11) ewes. The values presented in the figure are mean (±SD) concentrations in plasma collected monthly every 15 min for 2 h. * – statistically significant difference between S and T (P-value ≤ 0.05); a, b, c – mean values in a particular month marked with different letters differ significantly (P-value ≤ 0.05)

As it was in the case of KiSS-10, the plasma NKB concentration also generally trended upwards with the month of life (Fig. 2). In the singletons, the concentrations of this neuropeptide increased from the fourth to eighth months. The month-eight value for this group was significantly higher (P-value ≤ 0.05) than the values for the other months and was 384.19 ng/L. In the twins, increases in the plasma level of NKB were also observed from the fourth to ninth months, with the maximum of 364.70 ng/L coming in the ninth month of life. Up to the end of month eight, the NKB level was higher in S ewe lambs than in T animals. Only in the 9^th^ and 10^th^ months was an inverse relationship observed. The times of the highest NKB concentrations in blood plasma correlated with the initiation of ovarian activity in both groups. A positive correlation was shown between the changes in the plasma NKB concentration and age for all ewes (between the fourth and eighth months of life) (r = 0.79, P ≤ 0.05). There was a positive relationship between the NKB concentration and the age (r = 0.60 and r = 0.87 for S and T, respectively) of the ewes. There was a significant (P ≤ 0.05) difference in plasma NKB between the groups after the completion of the eighth month of life.

### Concentration of dynorphin A in blood plasma

**Fig. 3.**
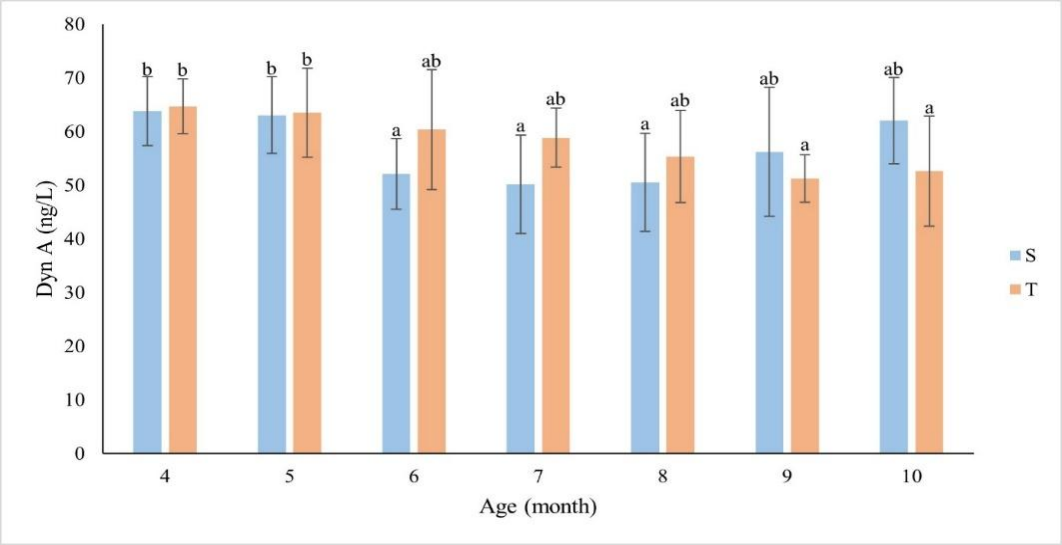
Concentration of dynorphin A (Dyn A) (ng/L) by month of life of singleton (S – n = 13) and twin (T – n = 11) ewes. The values presented in the figure are mean (±SD) concentrations in the plasma collected monthly every 15 min for 2 h. a, b – mean values obtained in a particular month marked with different letters differ significantly (P-value ≤ 0.05)

There was an opposite relationship between the Dyn A concentration and ewe lamb age to that noted for KiSS-10 and NKB (Fig. 3). The highest Dyn A concentrations were observed in the fourth and fifth months in both the singletons and the twins. In S group animals, a significant decrease (P ≤ 0.05) was observed between the fifth and sixth month, and the lowest levels were recorded in the seventh and eighth months of life. In T ewes, the mean Dyn A concentration decreased much more slowly, reaching the lowest value in the ninth month. Contrary to the KiSS-10 and NKB concentrations, those of Dyn A were lower in group S than in group T between the fourth and eighth months. Only in the 9^th^ and 10^th^ months were the S Dyn A concentrations higher than the T ones, and in the 10^th^ month they regained their 4^th^- and 5th-month levels. There was an inverse relationship between the Dyn A concentration and the age (r = -0.26 and r = -0.97 for S and T, respectively) of the ewes. No statistically significant differences were found between S and T concentrations in particular months of life. There was also no significant relationship between the changes in the plasma Dyn A concentration in S and T with age (r = 0.30).

### Relationship between KiSS-10, NKB and Dyn A concentrations in ewes coming from single and twin pregnancies

An overall positive correlation was found between the changes in the KiSS-10 and NKB plasma concentrations with age (between the 4^th^ and 10^th^ months of life) in both S and T (Table 2). However, an overall negative correlation was shown between KiSS-10 and Dyn A and between NKB and Dyn A over the seven studied months in all ewes.

**Table 2.** Pearson correlation coefficients (r) (P ≤ 0.05) between plasma concentrations of KiSS-10, NKB and Dyn A in singleton and twin Polish Uhruska ewes between their 4^th^ and 10^th^ months of life

		KiSS-10	NKB	Dyn A
	KiSS-10	–	0.86	–0.55
Singletons	NKB	0.86	–	–0.89
	DynA	–0.55	–0.89	–
	KiSS-10	–	0.89	–0.98
Twins	NKB	0.89	–	–0.94
	DynA	–0.98	–0.94	–

**Fig. 4.**
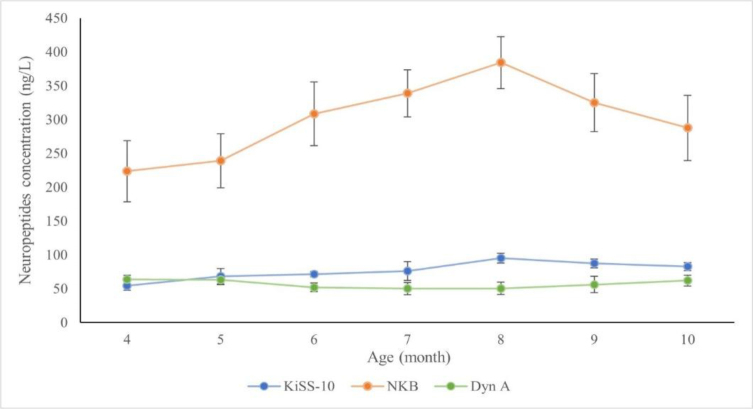
Changes in plasma kisspeptin-10 (KiSS-10), neurokinin B (NKB) and dynorphin A (Dyn A) concentrations in singleton Polish Uhruska ewes from the 4^th^ to 10^th^ month of life

**Fig. 5.**
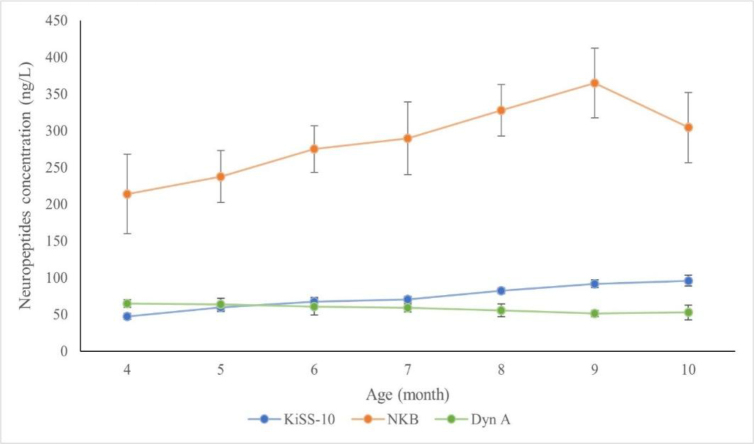
Changes in plasma kisspeptin-10 (KiSS-10), neurokinin B (NKB) and dynorphin A (Dyn A) concentrations in twin Polish Uhruska ewes from the 4^th^ to 10^th^ month of life

A graphical summary of changes in the blood plasma concentrations of individual neuropeptides in the singletons and the twins is presented in Figs 4 and 5.

## Discussion

Puberty is a major multifactorial phenomenon that involves neuroendocrinological changes leading to the onset of sexual maturity in animals and humans. A key step in initiating this process is an increase in pulsatile secretion of GnRH and gonadotropins that promotes the maturation of ovarian follicles, leading to elevated oestrogen levels that initiate the GnRH and LH surge resulting in the occurrence of the first ovulation. The onset of puberty in sheep can be delayed by genetic and nutritional factors, environmental conditions and hormonal and metabolic regulation (1, 5, 24, 28). A low birth weight is one of the factors contributing to delayed puberty in lambs, particularly in those born from twin pregnancies or to ewes with a high body weight (9, 22, 23). In general, ewes reach puberty once they attain a critical body weight and a metabolic condition that enables the hypothalamic–pituitary–gonadal (HPG) axis to become fully functional. Any disturbance in these processes can interfere with the normal timing of sexual maturation and delay the onset of reproductive function. The specific neural processes responsible for the rise in GnRH and secretion of gonadotropins at the onset of puberty are not fully elucidated. However, it is known that three neuropeptides seem to play a key role in this process: KiSS, NKB and Dyn produced by KNDy neurons in the ARC of the hypothalamus (30) and the anterior pituitary (27).

The study of anterior pituitary cells collected from pubescent ewes noted their LH and FSH secretion to be potentiated by KiSS-10 *in vitro* (27). This suggests the potential role of KiSS in gonadotropin secretion not only *via* mechanisms in the hypothalamus but also directly at the pituitary level. The hypothalamic contribution has been extensively documented. In sheep, the *KISS1* gene’s expression in the ARC has been shown to increase significantly in the period preceding the first ovulation (21). In addition, intravenous administration of kisspeptin to prepubertal ewe lambs increased the LH pulse frequency, indicating its ability to activate the reproductive axis despite the immaturity of the neuroendocrine system (21). The importance of kisspeptin has also been clearly confirmed in studies in other species. Mice with knocked out *KISS1* or *GPR54* (the gene encoding the orphan G protein-related membrane receptors for KiSS) did not enter puberty and developed permanent hypogonadism (10). In humans, mutations in the *GPR54* gene cause congenital hypogonadotropic hypogonadism, leading to delayed or completely suppressed puberty (6). In primates such as *Macaca mulatta*, KiSS-10 administration also activated pulsatile GnRH and LH secretion, indicating that the mechanism of action of this neuropeptide is conserved among mammals (16). Studies conducted in juvenile monkeys showed that repeated intravenous administration of KiSS-10 led to premature GnRH secretion, which in turn initiated sexual maturation. All these observations support the idea that KiSS-10 plays a primary role in the regulation of the HPG axis, and its deficiency or any disruption of the KISS1/GPR54 signalling pathway may be one of the key pathogenic links in cases of delayed puberty (16, 19). In the present study, we have shown that the plasma KiSS-10 levels in ewes coming from single pregnancies increased progressively from the fourth to the eighth months of life, reaching the highest level in the study period in the eighth month. In contrast, the KiSS-10 concentrations rose in the ewes from multiple pregnancies throughout the study. The highest plasma KiSS-10 concentration in this group was recorded after the 10^th^ month. These results correlated with the initiation of ovarian activity, which was monitored laparoscopically in both groups. The later peak of the KiSS-10 level in the ewes coming from twin pregnancies could be related to delayed puberty; however, precise determination of the time and mechanism of ovine achievement of sexual maturity will require investigation of GnRH/LH pulses in further research studies. As reported by Yang *et al*. (35), higher average plasma kisspeptin levels were observed in a group of girls with idiopathic central precocious puberty than in a group with simple premature thelarche and the control group. In summary, plasma KiSS levels increase at the onset of puberty, which suggests that this neuropeptide may serve as a marker to assess the effectiveness of treatment for precocious puberty.

Neurokinin B can stimulate GnRH secretion *via* the NK3R receptor. Studies using the NK3R agonist senktide in prepubertal lambs have shown an increase in LH release, confirming the functional role of NKB in modulating GnRH activity even before sexual maturity (14). Also, studies in adult female sheep have shown that activation of the NK3R receptor by this agonist stimulates LH secretion, indicating that NKB plays an important role in the proper functioning of the HPG axis (4). Importantly, NK3R receptors are not present on GnRH neurons themselves but are abundantly expressed in hypothalamic regions, such as the ARC, POA and particularly the retrochiasmatic area. The stimulatory effect of NKB on GnRH secretion is thus mediated indirectly, most likely through KNDy neurons located in the ARC. These neurons co-express KiSS and NKB, which are essential for the generation of pulsatile GnRH release (4). In our current study, we have demonstrated that the plasma concentration of NKB, similarly to that of KiSS-10, was dependent on the month of age. In the singletons, the NKB levels gradually increased between the fourth and eighth months of life, with the highest concentration in the whole study period observed in the last of these months. In the twins, comparable rises in plasma NKB levels were noted from the fourth to the ninth months, reaching the mean highest level during the entire observed period in the ninth month of life. The later highest secretion of NKB by the twins may be associated with the delayed puberty in this group. In addition to these hypothalamic mechanisms, NKB also exerts direct effects at the pituitary level. In our previous *in vitro* study, NKB caused an increase in KiSS-10 secretion by anterior pituitary cells collected from pubescent ewes (26). This suggests a potential direct role of NKB in the secretion of KiSS-10 at the pituitary level. We have also shown that the *in vitro* exposure of ovine pituitary cells to NKB led to an increase in gonadotropin secretion (27). Therefore, the increase in NKB observed at the onset of puberty may be due not only to regulatory mechanisms in the hypothalamus but also to mechanisms related to the pituitary gland.

Available data indicate that Dyn A acts as an inhibitor on KNDy neurons *via* the KOR, reducing the frequency of GnRH pulses and, consequently, decreasing gonadotropin secretion (15). Weems *et al*. (32) provided functional evidence for the involvement of dynorphin in the termination of the GnRH pulse in sheep. In addition, studies conducted in prepubertal Suffolk sheep indicated the potential role of Dyn A in delaying puberty by inhibiting the pulsatile secretion of LH. Central administration of norbinaltorphimine (a KOR antagonist) increased LH secretion in ovariectomised prepubertal Suffolk ewe lambs treated with 17β-oestradiol, but not in the same sheep after reaching puberty (12). Studies conducted in other animal species showed that dynorphin inhibited pulsatile LH secretion: in prepubertal female Wistar-Imamichi rats, the neuropeptide delayed puberty, and chronic intraperitoneal administration of norbinaltorphimine resulted in an increased LH pulse frequency *via* attenuation of KOR signalling in KNDy neurons in the ARC (13). As shown by Harlow *et al*. (7), the number of ARC cells expressing dynorphin mRNA was lower in postpubertal gilts than in the prepubertal period. However, studies conducted on Corriedale ewes showed that the quantity of ARC cells expressing dynorphin mRNA was higher during the postpubertal luteal phase (11). The results of our current study showed that the level of Dyn A in blood plasma reached the lowest value in the seventh month of life in ewes coming from single pregnancies and in the ninth month of life in the case of sheep coming from twin pregnancies and thereby predisposed to delayed puberty. Considering the inhibitory effect of Dyn A on the pulsatile secretion of GnRH, which postpones the onset of puberty, the decrease in the level in the months in which the highest levels of KiSS-10 and NKB were observed seems to be correlated with the onset of ovarian activity in both groups. The highest plasma Dyn A concentrations in the singletons and the twins was observed in the fourth and fifth months, before the animals reached sexual maturity. Therefore, in the present study, we observed an inverse relationship between the Dyn A concentration and the age of ewes, compared to KiSS-10 and NKB. Moreover, in our previous *in vitro* research Szysiak *et al*. (27), we demonstrated that Dyn A increased LH and FSH secretion by anterior pituitary cells from pubescent ewe lambs. As reported in the earlier study Szysiak *et al*. (26), Dyn A had no significant effect on KiSS-10 secretion by ovine pituitary cells *in vitro*. This may suggest that Dyn A stimulates gonadotropin secretion directly at the pituitary level.

In the present study, a positive correlation was shown between the changes in the KiSS-10 plasma concentrations and those in the NKB concentrations persisting from the 4^th^ to the 10^th^ month of life in ewes coming from both single and twin pregnancies. However, a negative correlation was found between the KiSS-10 and the Dyn A concentrations and between those of NKB and of Dyn A in the same frame of reference. It appears that the initiation of ovarian activity through the occurrence of the first ovulation is associated in ewes of both birth circumstances with concurrent positively correlated increases in KiSS-10 and NKB concentrations with age in the blood plasma, and decreases in the Dyn A concentration. In our previous *in vitro* study, a high positive correlation was observed between the secretion of NKB and that of KiSS-10 by anterior pituitary cells isolated from pubescent ewes (26). Therefore, NKB modulates KiSS-10 secretion at the pituitary level, with its impact depending on the time of exposure and its concentration in the culture medium (26). Although the direct effect of NKB on *in vitro* KiSS-10 secretion by ovine pituitary cells does not directly correspond to KiSS-10 levels in blood plasma, a comparable interaction might take place in other locations where these neuropeptides are produced. Our previous study’s results point to Dyn A having no significant effect on the secretion of KiSS-10 by pituitary cells in pubescent ewes (26). Therefore, the observed inverse relationship between changes in KiSS-10 and Dyn A plasma concentrations may have been caused by other factors. The elucidation of the relationship between KiSS-10, NKB and Dyn A, especially the inverse correlation between KiSS-10 and Dyn A and between NKB and Dyn A, requires further research.

## Conclusion

In the present study, we characterised the relationship between KiSS-10, NKB and Dyn A in the course of normal and delayed puberty in pubescent ewes from the 4^th^ to the 10^th^ month of life. Our findings suggest that these neuropeptides may regulate the timing of reproductive activity onset in sheep. Further studies are needed to elucidate the specific roles of KiSS-10, NKB and Dyn A in regulating the HPO axis during sexual maturation or in the mechanisms underlying delayed puberty in ewes.
